# Traditional Chinese Medicine Combined With Chemotherapy and Cetuximab or Bevacizumab for Metastatic Colorectal Cancer: A Randomized, Double-Blind, Placebo-Controlled Clinical Trial

**DOI:** 10.3389/fphar.2020.00478

**Published:** 2020-04-21

**Authors:** Ningning Liu, Chaojun Wu, Ru Jia, Guoxiang Cai, Yan Wang, Lihong Zhou, Qing Ji, Hua Sui, Puhua Zeng, Haijuan Xiao, Huaimin Liu, Jiege Huo, Yuanyuan Feng, Wanli Deng, Qi Li

**Affiliations:** ^1^ Department of Medical Oncology, Shuguang Hospital, Shanghai University of Traditional Chinese Medicine, Shanghai, China; ^2^ Academy of Integrative Medicine, Shanghai University of Traditional Chinese Medicine, Shanghai, China; ^3^ Department of Colorectal Cancer Surgery, Fudan University Shanghai Cancer Center, Shanghai, China; ^4^ Cancer Research Institute, Hunan Academy of Traditional Chinese Medicine, Changsha, China; ^5^ Department of Oncology, Hospital Affiliated to Shaanxi University of Chinese Medicine, Xianyang, China; ^6^ Department of Integrated Chinese and Western Medicine, Henan Provincial Cancer Hospital, Affiliated Tumor Hospital of Zhengzhou University, Zhengzhou, China; ^7^ Department of Oncology, Affiliated Hospital of Integrated Traditional Chinese and Western Medicine, Nanjing University of Chinese Medicine, Nanjing, China

**Keywords:** traditional Chinese medicine, metastatic colorectal cancer, cetuximab (CET) or bevacizumab (BV), progression-free survival, quality of life

## Abstract

**Background:**

Huangci Granule is a traditional Chinese medicine for treating metastatic colorectal cancer (mCRC).

**Objective:**

To evaluate the efficacy and safety of Huangci Granule combination with chemotherapy and cetuximab (CET) or bevacizumab (BV) for treating mCRC.

**Methods:**

We performed a randomized, controlled, and double-blind trial and recruited patients with mCRC who were planned to undergo chemotherapy combined with CET or BV. The treatment group was treated with Huangci Granule, while the control group was treated with placebo. Continuous treatment until disease progression, death, intolerable toxicity or up to 6 months. The primary endpoint was progression-free survival (PFS), and the secondary endpoint was quality of life and safety.

**Result:**

320 patients were randomly assigned to receive treatment, including 200 first-line patients and 120 second-line patients. In the first-line treatment, the median PFS was 9.59 months (95% CI, 6.94–13.25) *vs* 6.89 months (95% CI, 4.99–9.52) in treatment group and control group (HR, 0.69; 95% CI, 0.50–0.97; *P* = 0.027). Chinese medicine was an independent factor affecting the PFS. In the second-line treatment, the median PFS was 6.51 months (95% CI, 4.49–9.44) *vs* 4.53 months (95% CI, 3.12–6.57) in the treatment group and control group (HR, 0.65; 95% CI, 0.45–0.95; *P* = 0.020). Compared with the control group, “role function,” “social function,” “fatigue,” and “appetite loss” were significantly improved in the treatment (*P* < 0.05) and drug related grades 3 to 4 adverse events were less.

**Conclusion:**

Huangci Granule combined with chemotherapy and CET or BV can prolong the PFS of mCRC, improve the quality of life, reduce adverse reactions, and have good safety.

## Introduction

Colorectal cancer is one of the most common malignant tumors of digestive tract. The global cancer statistics report of 2018 shows that the incidence of colorectal cancer ranks third in all malignant tumors and the mortality rate ranks second (Bray et al., 2018). The number of new cases and deaths of colorectal cancer in China ranks first in the world. In 2014, there were about 2,708,000 new cases and 1,313,000 deaths of colorectal cancer in China, which is much higher than that in other countries and regions (Chen et al., 2018). The 5-year survival rate of early colorectal cancer was more than 90%, while that of metastatic colorectal cancer was less than 14% (Ferlay et al., 2013; Siegel et al., 2018), the prognosis was very poor (Poston et al., 2008; Sousa et al., 2019).

The National Comprehensive Cancer Network Guideline recommends chemotherapy combined with CET or BV as a first-line treatment for metastatic colorectal cancer (Eichelmann et al., 2019). Since the approval of targeted drugs in the early 21st century, a number of large clinical studies have shown that the overall survival (OS) of patients with metastatic colorectal cancer has increased by nearly one year compared with chemotherapy alone (Modest et al., 2015; Venook et al., 2017). Tailor’s study for Chinese patients showed that the mPFS of patients with advanced RAS wild-type colorectal cancer after first-line CET treatment was 9.2 months, and the OS was 20.7 months (Qin et al., 2018). Targeted combined use has significantly prolonged the survival of patients with advanced colorectal cancer. However, its efficacy still needs to be further improved, and the toxicity of chemotherapy seriously affects the quality of life of the patients. Progress of disease due to drug resistance or adjust treatment due to intolerance is still the biggest factor limiting the efficacy (Lalla et al., 2014; Geng et al., 2017). Therefore, how to further prolong the survival time of patients with metastatic colorectal cancer, reduce adverse reactions and improve the quality of life is the urgent problem to be solved.

In China, Chinese herbal medicine has been used for more than 2000 years and has been widely used in cancer treatment, showing very little toxicity or side effects (Hsiao and Liu, 2010). For example, in a phase I/II clinical trial, Huangqin Decoction can reduce gastrointestinal toxicity induced by irinotecan, a chemotherapeutic drug (Lam et al., 2010). However, the absence of strong evidence-based research and the lack of standardization of the herbal products are the main obstacles toward the globalization of TCM (Hsiao and Liu, 2010). In our previous clinical practice, we found that the treatment of reinforcing kidney, removing toxic, eliminate the mass, and relieve swelling can effectively inhibit the metastasis of colorectal cancer after operation, and summed up the prescription of Bushen Jiedu Sanjie Formula, also known as Huangci Granule (produced by Jiangyin Tianjiang Pharmaceutical Co., Ltd. of China. batch number: 1610345), which consists of *Ligustrum lucidum*, 15 g; *Cistanche deserticola*, 12 g; snakeberry, 15 g; edible tulip, 15 g; *Salvia miltiorrhiza* Bge, 15 g; and Fruit of Fiverleaf Akebia, 9 g. Animal experiments have proved that the Chinese medicine compound can inhibit the growth of tumor cells (Feng et al., 2017), which provides evidence for clinical trials. By using the method of ultrahigh performance liquid chromatography-mass spectrometry (UPLC-Q-TOF/MS), 40 compounds were identified from the traditional Chinese medicine formula granules through database comparison and reference (see **Supplementary Material** for details.)

In order to further verify the clinical efficacy of the compound, we conducted a randomized controlled double-blind trial to evaluate the efficacy and safety of the combination of chemotherapy and targeted therapy in patients with metastatic colorectal cancer.

## Patients and Methods

### Research Design

This study is a randomized, double-blind, placebo-controlled clinical trial. A total of 320 people were randomly divided into treatment group and control group (1:1). On the basis of chemotherapy and CET or BV, two groups of patients were treated with Chinese herbal compound or placebo twice a day until the disease progressed, death, intolerable toxicity or up to 6 months. This study has been registered in the Chinese Clinical Trial Registry (ChiCTR), registration No. ChiCTR-IOR-16008843 (registration date: 2016-07-14).

### Subjects

#### Entry Criteria

1. Patients with stage IV colorectal cancer had definite cytological or pathological diagnosis;
2. Patients will receive first line chemotherapy combined with targeted therapy (the first treatment or the interval from the last chemotherapy was more than 6 months). Or the patient has been treated with first-line chemotherapy alone, and the disease has progressed. Now it is proposed to be treated with chemotherapy and CET or BV for the first time, and the expected survival period is more than 6 months.
3. At least one measurable lesion displayed by imaging examination (PET-CT, CT, MRI, bone scan, X-ray);
4. Those who accord with the diagnosis of Kidney Deficiency Syndrome and Damp-heat Syndrome in TCM;
5. The age ranged from 18 to 75 years, and the body condition score was ECOG (0-1);
6. The blood routine was normal, the function of heart, liver, and kidney was not abnormal, and the electrocardiogram was basically normal;
7. The patient has good compliance, can understand the situation of this study and sign the informed consent.

#### Exclusion Criteria

1. There are serious diseases of cardiovascular system, urinary system, blood system, and digestive system, which cannot be tolerated by experimental researchers;
2. Pregnant or lactating women with uncontrollable mental disorders;
3. Complicated with active tuberculosis and other serious infectious diseases;
4. Those who cannot take oral medicine or vomit frequently;
5. Those with poor compliance;
6. In the past month, other trial drugs or patients in other clinical trials were used.

### Diagnosis of Kidney Deficiency Syndrome and Damp-Heat Syndrome

The diagnosis of TCM Syndromes of patients was completed by two experienced TCM physicians at baseline evaluation. Main symptoms: weakness of waist and knee, hyposexuality, chilly sensation and the cold limbs, feverish sensation over the five centers (palms, soles, and chest), abdominal fullness and distention, thirst and less drinking, pale tongue, greasy, and yellow fur on the tongue, deep and thin pulse, etc.

#### Interventions

Subjects were given Huangci Granule/placebo on the basis of chemotherapy and targeted therapy, 12 g per bag, twice a day, after breakfast and dinner. Huangci Granule and placebo are produced by Jiangyin Tianjiang Pharmaceutical Co., Ltd. of China. The placebo consists of bitter compounds and excipient. According to NCCN guidelines, the chemotherapeutic regimen was recommended by the physician to choose mFOLFOX6/FOLFIRI/CAPEOX or other regimen, and appropriate dosage adjustment was made when patients developed intolerance. According to the results of RAS and BRAF gene detection, BV (5 mg/kg, intravenous infusion, 1 day, 2 weeks repeat) or CET (500 mg/m2, intravenous infusion, 1 day, 2 weeks repeat) can be used in patients with wild type RAS gene and BRAF gene. Mutants can only use BV.

#### Randomization

The subjects were randomly assigned to two groups, with a ratio of 1:1. Randomized according to a random sequence table (generated by SAS 8.2). The randomly assigned treatment was sealed in opaque envelopes and controlled by designated personnel who were not involved in the trial.

#### Blind Method

In order to ensure the success of the double-blind test, the drug simulation test was carried out. The appearance, shape, color and packaging of Chinese medicine compound placebo are the same as those of drugs. In order to ensure the authenticity of the statistical results and final reports, blind methods were used for both researchers and patients. Blindness was revealed after all the data were collected or serious adverse events occurred.

#### Outcome Assessment

The main outcome was PFS from the start of the trial to the first progression or death. According to RECIST 1.1 standard, patients were assessed every two treatment cycles. Patients were followed up every 1 month until the disease progressed or died.

Secondary outcomes include quality of life and safety. The safety assessment was based on Common Terminology Criteria Adverse Events Version 4.0 (CTCAE v4.0), and the quality of life score was based on the European Organization for Research on Treatment of Cancer (EORTC) Quality of Life Scale QLQ-C30 v3.0. Safety assessment was conducted before admission and the start of each treatment cycle. Once adverse events occurred, they were recorded immediately and observed until 1 month after discharge. The EORTC QLQ-C30 scale was used to assess the quality of life in patients with symptoms, functions, and living conditions by linear conversion to percentage system.

### Statistical Analysis

SPSS 21.0 software was used for data analysis. Using t test or Wilcoxon rank sum test to compare age and other measurement data, using chi-square test or Fisher exact test to compare gender, primary tumor location, and metastasis count data, and to compare the baseline level. Kaplan Meier method was used for survival analysis. Cross-analysis, rank sum test, and Cox regression model were used to analyze the related factors. All statistical tests are unilateral, and P value less than 0.05 is defined as having statistical difference.

## Results

**Figure 1:**
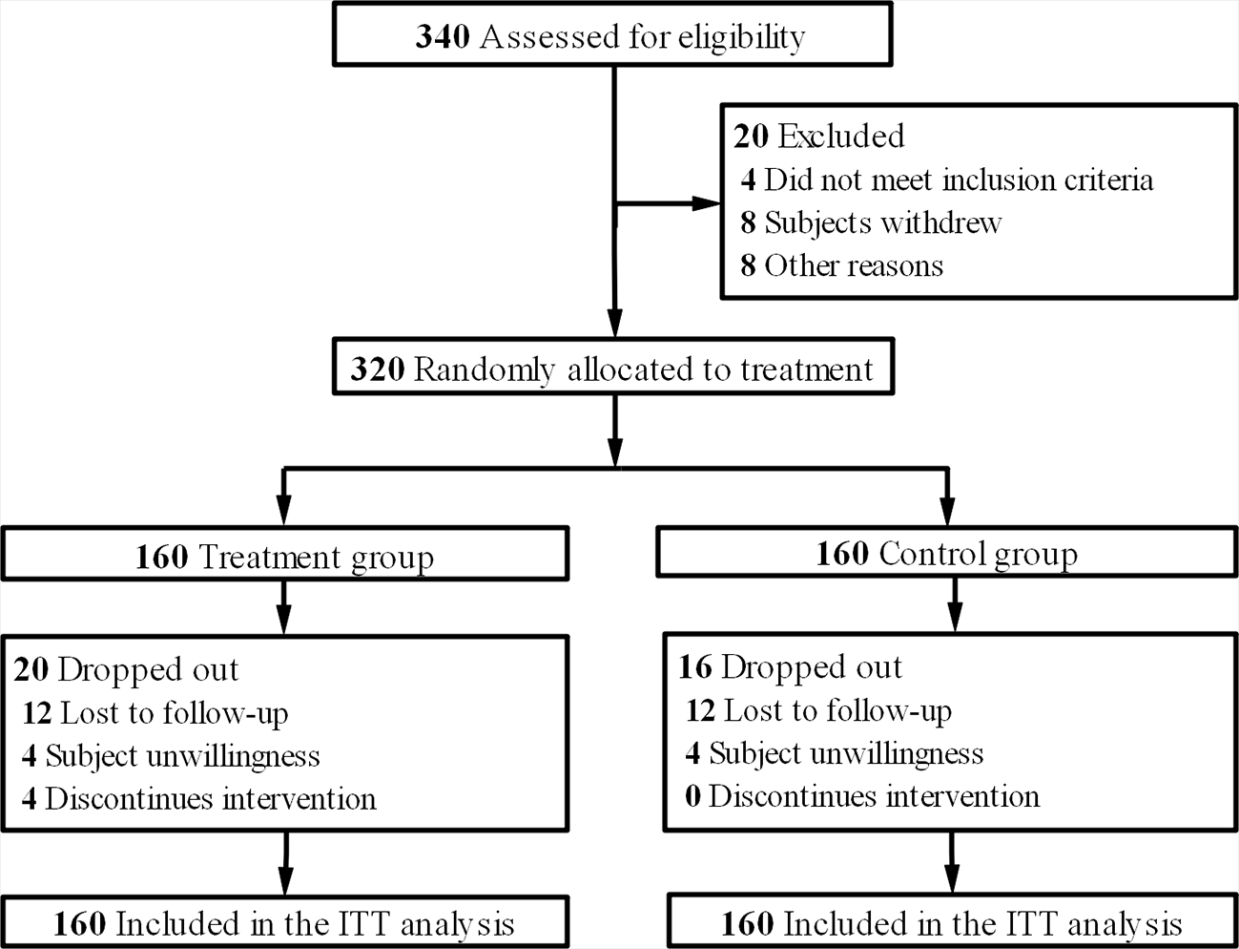
Trial profile.

From August 2016 to June 2018, 340 eligible patients were screened. These patients came from Department of Medical Oncology, Department of Gastrointestinal Surgery, Shuguang Hospital, Shanghai University of Traditional Chinese Medicine; Department of Colorectal Cancer Surgery, Fudan University Shanghai Cancer Center; Department of Medical Oncology, Hunan Academy of Traditional Chinese Medicine Affiliated Hospital; Department of Oncology, Hospital Affiliated to Shaanxi University of Chinese Medicine; Department of Integrated Chinese and Western Medicine, Henan Provincial Cancer Hospital, Affiliated Tumor Hospital of Zhengzhou University and Department of Oncology, Affiliated Hospital of Integrated Traditional Chinese and Western Medicine, Nanjing University of Chinese Medicine. After eliminating 20 people for various reasons, they entered the randomization stage. The final analysis data were from 160 patients in the treatment group and 160 in the control group according to the intention-to-treat principle (**Figure 1**). The data deadline for the current analysis is April 30, 2019. The baseline characteristics of the two groups were studied, and the distribution of demographic and clinical characteristics between the two groups was balanced (**Table 1**).

**Table 1:** Baseline characteristics.

Characteristic	Huangci Granule + CT+ BV/CET group (n = 160)	Placebo+ CT+ BV/CET group (n = 160)
Age (years)	63.89 ± 10.11	62.19 ± 7.84
<65	73 (46%)	89 (56%)
≥65	87 (54%)	71 (44%)
Gender		
Male	87 (54%)	111 (69%)
Female	73 (46%)	49 (31%)
ECOG performance status		
0	82 (51%)	93 (58%)
1	78 (49%)	67 (42%)
Disease stage at diagnosis		
II	14 (9%)	22 (14%)
III	64 (40%)	49 (31%)
IV	82 (51%)	89 (55%)
Primary tumor location		
Left	119 (74%)	129 (81%)
Right	41 (26%)	31 (19%)
Metastasis		
1	117 (73%)	93 (58%)
≥2	43 (26%)	67 (42%)
Chemotherapy		
Based on fluoropyrimidines	9 (6%)	4 (3%)
Based on oxaliplatin	73 (46%)	76 (47%)
Based on irinotecan	78 (49%)	80 (50%)
Targeted therapy		
Bevacizumab	128 (80%)	111 (69%)
Cetuximab	32 (20%)	49 (31%)

### Progression-Free Survival

**Figure 2:**
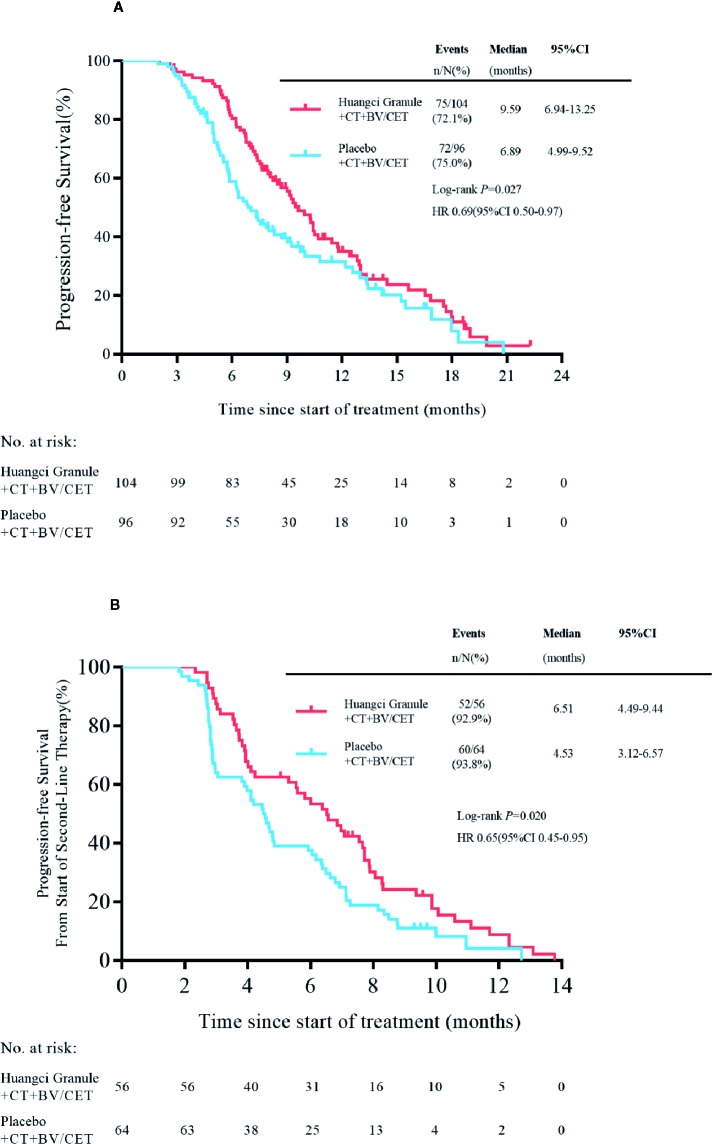
Kaplan-Meier survival estimates for progression-free survival. **(A)** first-line. **(B)** second-line.

In the first-line treatment, the PFS of the treatment group was significantly longer than that of the control group. The mPFS was 9.59 months (95% CI, 6.94–13.25) in the treatment group and 6.89 months (95% CI, 4.99–9.52) in the placebo group (HR, 0.69; 95% CI, 0.50–0.97; *P* = 0.027) (**Figure 2A**).

**Figure 3:**
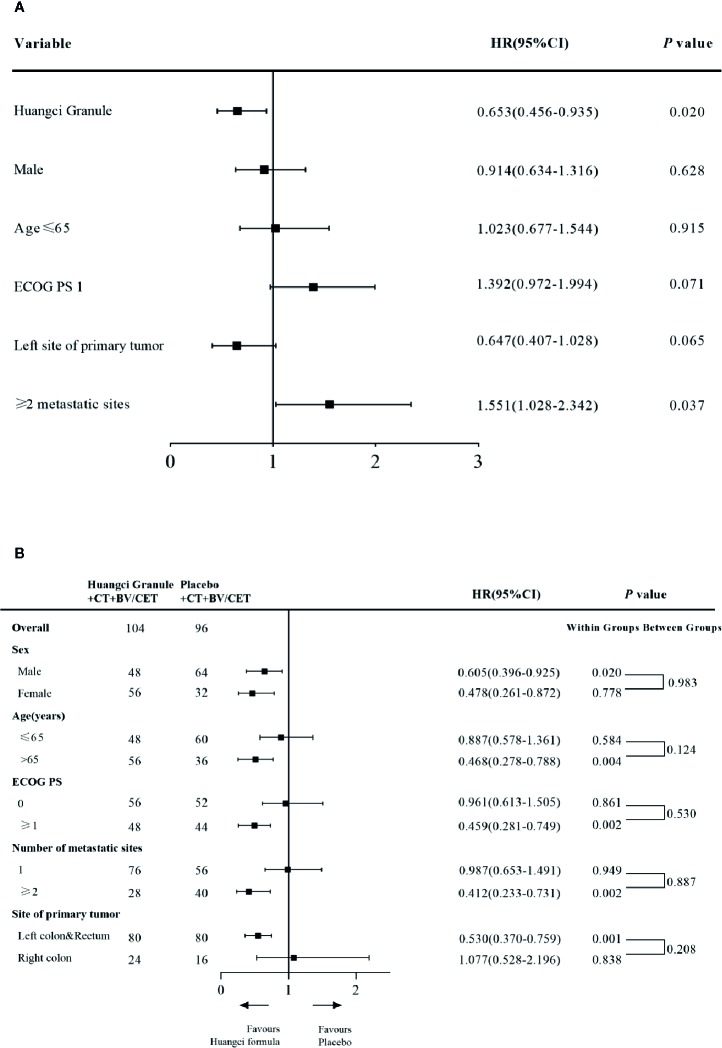
**(A)** Cox and subgroup analysis for PFS Univariate Cox proportional hazards regression analysis of PFS **(B)** Subgroup analysis for PFS.

Cox regression model was used to study the effect of prognostic factors on PFS. It was found that the combination of traditional Chinese medicine compound has a stable protective effect on PFS, and multiple metastases are a poor prognostic factor (**Figure 3A**). By subgroup analysis, all the subgroups except right colon patients showed that the combination of Chinese herbal medicine could benefit the progression-free survival of patients more (**Figure 3B**). In the second-line treatment, the mPFS of the treatment group was 6.51 months (95% CI, 4.49–9.44), while that of the control group was 4.53 months (95% CI, 3.12–6.57) (HR, 0.65; 95% CI, 0.45–0.95; *P* = 0.020) (**Figure 2B**), and the Chinese herbal compound could also improve the progression-free survival.

### Quality of Life

**Figure 4:**
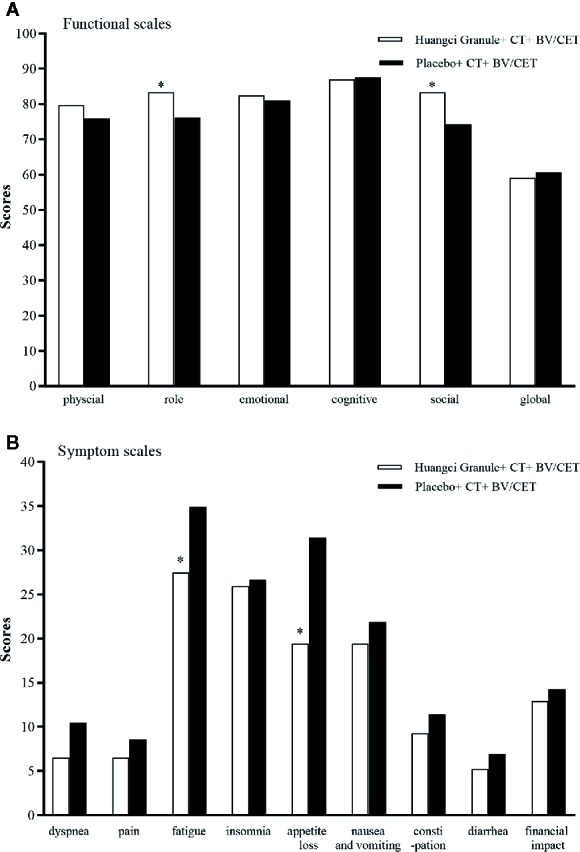
Quality of life scores between the two groups. *P < 0.05. **(A)** Functional scales. **(B)** Symptom scales.

QLQ-C30 can comprehensively evaluate the quality of life in multiple latitudes. In EORTC QLQ-C30 baseline level, there is no significant difference in the mean values of multiple items and single items between the two groups, which is comparable (**Table 2**). In terms of function, the level of role function (83.33 ± 13.21 vs 76.19 ± 13.58) and social function (83.33 ± 13.80 vs 74.29 ± 15.31) in the treatment group were significantly higher than those in the control group (**Figure 4A**). The symptoms of fatigue (27.47 + 14.42 vs 34.92 + 16.42) and appetite loss (19.44 + 23.06 vs 31.43 + 25.49) in the treatment group were significantly lower than those in the control group (**Figure 4B**).

**Table 2:** Descriptive statistic of the QLQ-C30 before treatment.

Items	Huangci Granule + CT+ BV/CET group (n = 160)		Placebo+ CT+ BV/CET group (n =1 60)		*P* value
	Mean score	SD	Mean score	SD
Functional scales					
Physical	79.63	16.25	76.00	15.94	0.35
Role	84.26	12.56	81.43	14.45	0.38
Emotional	82.41	13.33	80.95	14.93	0.67
Cognitive	87.03	13.87	87.62	12.36	0.85
Social	83.79	14.08	77.14	16.71	0.07
Global quality of life	59.03	16.83	60.71	17.92	0.68
Symptom scales					
Dyspnea	6.48	13.38	10.48	15.70	0.08
Pain	6.48	12.14	8.57	13.63	0.50
Fatigue	28.09	14.18	34.60	16.78	0.08
Insomnia	25.93	28.85	26.67	27.77	0.91
Appetite loss	20.37	24.27	19.05	18.59	0.80
Nausea and vomiting	19.44	30.21	21.90	30.19	0.73
Constipation	9.26	21.98	11.43	22.78	0.68
Diarrhea	6.94	12.20	5.23	11.97	0.55
Financial impact	12.96	16.48	14.29	16.74	0.74

### Security

During the trial, no serious adverse events occurred in either group, and the study was discontinued. Among 160 patients receiving TCM compound therapy, 32 (20%) received at least one dose adjustment (reduction, postponement), while 60 (42%) received placebo treatment had dose adjustment. The incidence of dose reduction in the treatment group was lower. The incidence of grade 3 or higher treatment-related adverse events (TRAEs) in TCM group was lower than that in control group, Leukopenia (25% vs 45%), Fatigue (13% vs 38%) and Appetite loss (15% vs 30%). It is worth noting that in the common side effects of BV on hypertension, the Chinese herbal compound group has a lower incidence (19% vs 40%) (**Table 3**).

**Table 3:** Summary of TRAEs.

Treatment-related Adverse events	Huangci Granule + CT+ BV/CET group (n=160)		Placebo+ CT+ BV/CET group (n=160)		*P* value
	Grade 1–2	Grade 3–4	Grade 1–2	Grade 3–4
Leukopenia	64 (40%)	40 (25%)*	60 (38%)	72 (45%)	0.014
Hepatotoxicity	36 (23%)	12 (8%)	48 (30%)	12 (8%)	0.535
Nausea	56 (35%)	20 (13%)	52 (33%)	28 (18%)	0.240
Diarrhea	32 (20%)	16 (10%)	24 (15%)	22 (14%)	0.152
Fatigue	48 (30%)	20 (13%)*	80 (50%)	60 (38%)	0.032
Appetite loss	52 (33%)	24 (15%)*	52 (33%)	48 (30%)	0.028
PN	32 (20%)	16 (10%)	36 (23%)	16 (10%)	0.784
HFS	27 (17%)	12 (8%)	30 (19%)	24 (15%)	0.182
Rash	19 (12%)	12 (8%)	17 (11%)	24 (15%)	0.096
Hair loss	8 (5%)	28 (18%)	24 (15%)	52 (33%)	0.306
Hypertension	37 (23%)	30 (19%)*	41 (26%)	64 (40%)	0.038
Bleeding	8 (5%)	12 (8%)	8 (5%)	16 (10%)	0.647

PN, peripheral neuropathy; HFS, hand-foot syndrome.*P < 0.05.

## Discussion

Since the 21st century, a large number of biological agents have been developed, and their further clinical benefits have been demonstrated by their combination with cytotoxic chemotherapy (Peeters and Price, 2012). Among them, two kinds of molecular targeting agents are most famous, one is anti-vascular endothelial growth factor antibody (including BV, aflibercept, ramucirumab, regorafenib), the other is epidermal growth factor receptor antibody (including CET, panitumumab). In clinical study of CALGB/SWOG 80405, the mPFS of patients with BV-based regimens increased to 10.8 months (Venook et al., 2014), which increased by 2 months compared with the V308 test with chemotherapy alone (Mitry et al., 2004). In addition, the continued use of BV after failure of first-line BV combined with chemotherapy can prolong the survival of patients with mCRC (Bennouna et al., 2013). Similarly, CET, an anti-EGFR drug, showed improved Objective remission rate (ORR) and prolonged OS in a number of trials (Cunningham et al., 2004; Sobrero et al., 2008). Initially, these biological agents were used in all patients with mCRC. As the knowledge of colorectal cancer deepened, colorectal cancer was divided into different molecular subtypes to judge prognosis (Dienstmann et al., 2017). For example, KRAS or NRAS mutation population could not obtain survival prolongation (Van Cutsem et al., 2015) from anti-EGFR drugs. This selection of molecular targeted drugs based on specific gene loci has made a significant step forward in the individualized and precise treatment of metastatic colorectal cancer. However, the efficacy of BV and CET remains to be further improved, and anti-vascular endothelial growth factor can cause a series of vascular-related adverse reactions. For example, vascular injury can lead to bleeding and wound healing difficulties, abnormal vasoconstriction and diastolic will be accompanied by hypertension, proteinuria, and other clinical manifestations (Hurwitz et al., 2004). Skin reaction is the most common adverse reaction in anti-EGFR treatment. 80% to 86% of patients developed papular rash after CET treatment (Štulhofer Buzina et al., 2016). Therefore, how to reduce its adverse reactions is also the key to improve drug tolerance and quality of life for patients.

Traditional Chinese medicine (TCM) has been widely used in China, but there is a lack of systematic clinical trials to verify its efficacy (Zhao et al., 2018). Previous studies have shown that long-term use of traditional Chinese medicine can improve the survival rate of patients with stage II-III colorectal cancer after radiotherapy and chemotherapy (Xu et al., 2017), but the combination of traditional Chinese medicine and chemotherapy, targeted treatment for advanced colorectal cancer has not been reported. Our results show that TCM compound can improve PFS and quality of life of patients with chemotherapy combined with targeted therapy, and reduce the incidence of adverse reactions. In terms of PFS, the first-line PFS of the treatment group reached 9.59 months, and the second-line PFS reached 6.89 months, which were significantly longer than those of the control group, and the curative effect was also better than the clinical data of Chinese patients. Subgroup analysis further confirmed the validity of TCM compound.

Assessment of quality of life can provide clinicians with important guidance in making decisions to maximize patients’ rehabilitation needs and treatment benefits (Conroy et al., 2003). The QLQ-C30 scale developed by EORTC was used to evaluate the quality of life of patients, which effectively reflected the overall clinical health status of patients (Uwer et al., 2011). The CHARTA-AIO 0209 trial at the 2017 ASCO Annual Meeting compared the effects of FOLFOXIRI combined with BV and FOLFOX combined with BV on the quality of life of patients with advanced colorectal cancer. The results showed that the main symptoms were lower digestive tract scores, including nausea and vomiting, appetite loss (Quidde et al., 2017). Previous studies have found that TCM compound can improve the quality of life of cancer patients and reduce the incidence of adverse reactions to chemotherapy (Qi et al., 2015). Targeted drugs have different adverse reactions from chemotherapy, such as bleeding, rash, hypertension (Small et al., 2014; Monjazeb and Wilson, 2017). The improvement of TCM on these symptoms is also under our observation.

The incidence of adverse events, such as leukopenia, fatigue, appetite loss, and hypertension, in the treatment group was significantly lower than that in the control group. Taking traditional Chinese medicine prescriptions improves the quality of life in the fields of roles, social functions, and symptoms of fatigue and appetite loss. Some studies have shown that *Cistanche deserticola* can increase the expression of erythropoietin and receptor mRNA, promote the proliferation of bone marrow hematopoietic stem cells, improve bone marrow suppression (Dai et al., 2011), and induce apoptosis of tumor cells through mitochondrial dependent pathway (Li et al., 2016). The extract of *Salvia miltiorrhiza* can effectively inhibit the proliferation, tubule formation and metastasis of human colorectal cancer cells by lowering HIF-1α levels and inhibiting the secretion of VEGF and bFGF (Sui et al., 2017). These results are helpful to explain the results observed in this study.

This study provides the first conclusive evidence that TCM compound could prolong the PFS of patients with metastatic colorectal cancer, improve the quality of life and reduce the incidence of adverse events. Our clinical trials have proved the effectiveness of traditional Chinese medicine in the treatment of tumor diseases, which will help to eliminate the misunderstanding of traditional Chinese medicine, increase its acceptance, promote better and wider use, and provide scientific basis for further development of traditional Chinese medicine theory. The clinically effective formulations confirmed in this study can guide the development of new drugs, which may be further refined for better formulation and research. However, the current study is limited small sample size, short follow-up time, and lack of data on long-term efficacy such as OS. In future trials, we will further expand the sample size to confirm the reliability of the treatment scheme and further explore the mechanism of traditional Chinese medicine combined with chemotherapy in the treatment of colorectal cancer.

## Data Availability Statement

All datasets generated for this study are included in the article/**Supplementary Material**.

## Ethics Statement

The studies involving human participants were reviewed and approved by IRB of Shuguang Hospital affiliated with Shanghai University of TCM. The patients/participants provided their written informed consent to participate in this study.

## Author Contributions 

QL, NL and CW contributed to the conception of the study and critical revision of the manuscript. RJ, GC, YW, LZ, QJ, HS, PZ, HX, HL, JH, YF, and WD contributed to conduction of the study. NL contributed to the analysis and interpretation of the data. NL and CW drafted the manuscript. All authors declared no conflict of interest and approved the final version of the manuscript for submission.

## Funding

The study was financially supported by the Key projects of the National Natural Science Foundation of China (81830120), three-year Plan of Action for the Development of Traditional Chinese Medicine in Shanghai (ZY(2018-2020)-CCCX-2003-03), New Frontier Technological Projects of Shanghai Shenkang Hospital Development Center (SHDC12015124) and Key Projects of Shanghai Science and Technology Commission (16401970500).

## Conflict of Interest

The authors declare that the research was conducted in the absence of any commercial or financial relationships that could be construed as a potential conflict of interest.
