# Supplementary material for: Traditional Chinese Medicine Combined With Chemotherapy and Cetuximab or Bevacizumab for Metastatic Colorectal Cancer: A Randomized, Double-Blind, Placebo-Controlled Clinical Trial
Source: Front Pharmacol. 2020 Apr 21;11:478. doi: 10.3389/fphar.2020.00478 (PMC7187887; doi:10.3389/fphar.2020.00478)
Supplement: Supplementary file 1 [file DataSheet_1.docx]

Component identification of Huangci granule

Figure 1 Schematic diagram of chromatographic peak of Huangci granule (positive ion mode)

Figure 2 Schematic diagram of chromatographic peak of traditional Huangci granule (negative ion mode)

Figure 3 Schematic diagram of ultraviolet absorption of Huangci granule (254 nm)

Figure 4 Schematic diagram of identification number of chromatographic peak of Huangci granule (positive ion mode)

Figure 5 Schematic diagram of identification number of chromatographic peak of Huangci granule (negative ion mode)

Figure 6 Schematic diagram of DADnumber of Huangci Huangci granule (254 nm)

Table 1 Identification results of chemical components

| Serial number | retention time（min） | Adduct ions | M/Zactual value | M/Z Theoretical value | ppm | Molecular formula | Chinese name | English name | MS/MS spectra | medicinal materials |
| --- | --- | --- | --- | --- | --- | --- | --- | --- | --- | --- |
| 1 | 0.82 | [M-H]- | 191.0571 | 191.0572 | 1.0 | C7H12O6 | 奎宁酸 | Quinic acid | 191.0571,127.0394,93.0345,85.0297 | Ligustrum lucidum |
| 2 | 3.21 | [M-H]- | 197.0455 | 197.0455 | 0.8 | C9H10O5 | 丹参素 | Danshensu | 197.034,135.0445,123.0444 | Salvia miltiorrhiza Bge |
| 3 | 3.88 | [M-H]- | 373.1140 | 373.1138 | -1.7 | C16H22O10 | 京尼平苷酸 | Geniposidic acid | 373.1107,211.0600,167.0704,123.0448 | Cistanche deserticola |
| 4 | 5.26 | [M-H]- | 375.1297 | 375.1303 | -0.5 | C16H24O10 | 8-表马钱酸 | 8-epi-loganic acid | 375.1312,213.0771,151.0762 | Cistanche deserticola |
| 5 | 5.60 | [M-H]- | 623.2193 | 623.2177 | -1.4 | C26H40O17 | / | Kankanoside F | 623.2213,511.0596,477.1630,461.1680,315.1047 | Cistanche deserticola |
| 6 | 5.80 | [M-H]- | 375.1297 | 375.1303 | -0.5 | C16H24O10 | 马钱酸 | loganic acid | 375.1272，213.0752，151.0751 | Cistanche deserticola |
| 7 | 6.34 | [M+H]+ | 303.0135 | 303.0140 | 0.2 | C14H6O8 | 鞣花酸 | Ellagic acid | 303.0135,275.0202,257.0086, | snakeberry |
| 8 | 6.35 | [M-H]- | 433.0988 | 433.0996 | 0.3 | C17H22O13 | 10-羟基硫苷 | / | 433.0984,389.1077,209.0444,165.0549 | Ligustrum lucidum |
| 9 | 8.84 | [M-H]- | 291.0146 | 291.0144 | -0.5 | C13H8O8 | 短叶苏木酚酸 | Brevifolincarboxylic acid | 247.0237,191.0344,145.0295 | snakeberry |
| 10 | 10.22 | [M-H]- | 637.1045 | 637.1046 | -0.8 | C27H26O18 | 木犀草素3',7-二-O-葡糖苷酸 | Luteolin-3',7-di-O-glucoside | 637.1051,461.0729,285.0396 | / |
| 11 | 10.97 | [M-H]- | 801.2459 | 801.2450 | 0.1 | C35H46O21 | / | Cistantubuloside | 801.2502,783.2395,621.2018,179.0354 | Cistanche deserticola |
| 12 | 12.01 | [M-H]- | 247.0248 | 247.0244 | -0.9 | C12H8O6 | 短叶苏木酚 | Brevifolin | 247.0227,219.0285,191.0336,145.0288 | snakeberry |
| 13 | 13.32 | [M-H]- | 785.2510 | 785.2514 | -0.3 | C35H46O20 | 松果菊苷 | Echinacoside | 785.2556,623.2222,477.1643,461.1677 | Cistanche deserticola |
| 14 | 14.89 | [M-H]- | 769.2561 | 769.2549 | 1.0 | C35H46O19 | 金石蚕苷 | Poliumoside | 769.2564,623.2196,443.1593,161.0235 | Cistanche deserticola |
| 15 | 15.52 | [M-H]- | 701.2298 | 701.2294 | -0.2 | C31H42O18 | 新女贞苷 | Neonuzhenide | 701.2304,539.1798,469.1371,437.1463 | Ligustrum lucidum |
| 16 | 16.35 | [M-H]- | 623.1981 | 623.1990 | 0.4 | C29H36O15 | 毛蕊花糖苷 | Acteoside | 623.2006,461.1661,315.1067,161.0237 | Cistanche deserticola |
| 17 | 16.44 | [M-H]- | 827.2615 | 827.2627 | -0.2 | C37H48O21 | 管花苷 A | Tubuloside A | 827.2577,725.2468,665.2263,623.2160 | Cistanche deserticola |
| 18 | 17.33 | [M-H]- | 685.2349 | 685.2351 | 1.3 | C31H42O17 | 特女贞苷 | Specnuezhenide | 685.2412,523.1863,453.1415,421.1503 | Ligustrum lucidum |
| 19 | 17.39 | [M-H]- | 623.1981 | 623.1991 | 1.1 | C29H36O15 | 异毛蕊花糖苷 | Isoacteoside | 623.2009,461.1681,161.0244,135.0449 | Cistanche deserticola |
| 20 | 17.53 | [M-H]- | 417.0827 | 417.0830 | 0.0 | C20H18O10 | 丹酚酸 D | Salvianolic acid D | 417.0813,373.0980,237.0389,219.0284 | Salvia miltiorrhiza Bge |
| 21 | 18.15 | [M-H]- | 685.2349 | 685.2373 | 0.7 | C31H42O17 | 异女贞子苷 | Isonuezhenide | 685.2345,523.1805,453.1404,299.1123 | Ligustrum lucidum |
| 22 | 20.06 | [M-H]- | 493.1140 | 493.1152 | 1.8 | C26H22O10 | 丹酚酸 A | Salvianolic acid A | 493.1133,313.0700,295.0596,185.0231, | Salvia miltiorrhiza Bge |
| 23 | 22.03 | [M-H]- | 717.1461 | 717.1481 | 1.4 | C36H30O16 | 丹酚酸 B | Salvianolic acid B | 717.1487,519.0944,339.0511,321.0399 | Salvia miltiorrhiza Bge |
| 24 | 22.34 | [M-H]- | 717.1461 | 717.1495 | 1.1 | C36H30O16 | 丹酚酸 E | Salvianolic acid E | 717.1495,519.0957,339.0517,321.0408 | Salvia miltiorrhiza Bge |
| 25 | 24.42 | [M-H]- | 283.0611 | 283.0612 | -0.7 | C16H12O5 | 芜花素 | Genkwanin | 283.0579,268.0364,211.0384 | edible tulip |
| 26 | 24.67 | [M-H]- | 1071.3562 | 1071.3570 | 2.4 | C48H64O27 | 女贞苷G13 | G13 | 1071.3646,839.2683,771.2414,685.2381,523.1683,453.1433 | Ligustrum lucidum |
| 27 | 25.28 | [M-H]- | 1089.5581 | 1089.5530 | 3.8 | C53H86O23 | / | Saponins PJ-1 | 1089.5375,601.1971,487.3395 | 预知子 |
| 28 | 25.81 | [M-H]- | 1071.3597 | 1071.3588 | -0.5 | C48H64O27 | 女贞苷G13异构体 | G13 isomer | 1071.3620,909.3037,839.2626,771.2388,685.2360,523.1823 | Ligustrum lucidum |
| 29 | 27.57 | [M-H]- | 1071.3597 | 1071.3588 | -0.8 | C48H64O27 | 女贞苷G13异构体 | G13 isomer | 1071.3707,909.3175,839.2660,771.2385,685.2390,523.1860 | Ligustrum lucidum |
| 30 | 27.72 | [M-H]- | 1071.3597 | 1071.3591 | -0.6 | C48H64O27 | 女贞苷G13异构体 | G13 isomer | 1071.3640,909.3175,839.2829,771.2409,685.2382,523.1848 | Ligustrum lucidum |
| 31 | 31.71 | [M-H]- | 1219.6152 | 1219.6156 | -0.3 | C59H96O26 | / | Saponins PK | 1219.6183,749.4464 | Fruit of Fiverleaf Akebia |
| 32 | 33.42 | [M-H]- | 1059.5381 | 1059.5393 | 1.7 | C52H84O22 | / | Saponins E | 1059.5318,735.4308,603.4140 | Fruit of Fiverleaf Akebia |
| 33 | 33.79 | [M-H]- | 1073.5538 | 1073.5544 | 2.4 | C53H86O22 | / | Saponins PJ-2 | 1073.5590,749.4526 | Fruit of Fiverleaf Akebia |
| 34 | 34.60 | [M-H]- | 927.4959 | 927.4962 | 1.3 | C47H76O18 | / | Saponins D | 927.5062,603.352 | Fruit of Fiverleaf Akebia |
| 35 | 39.15 | [M-H]- | 1043.5432 | 1043.5515 | 1.6 | C52H84O21 | / | Yuzhizioside Ⅳ | 1043.4716,719.4898,455.3594 | Fruit of Fiverleaf Akebia |
| 36 | 40.14 | [M-H]- | 911.5010 | 911.4989 | 0.2 | C47H76O17 | / | Pulsatilla Saponins D | 911.4986,749.4435,603.3999,471.3497 | Fruit of Fiverleaf Akebia |
| 37 | 40.48 | [M-H]- | 735.4325 | 735.4314 | 0.5 | C40H64O12 | / | Saponins B | 735.4380,603.3926,471.3517 | Fruit of Fiverleaf Akebia |
| 38 | 40.56 | [M-H]- | 749.4482 | 749.4482 | 0.7 | C41H66O12 | / | Saponins Pd | 749.4515,603.4011,471.3511 | Fruit of Fiverleaf Akebia |
| 39 | 40.94 | [M-H]- | 603.3902 | 603.3902 | -0.4 | C35H56O8 | / | Saponins A | 603.2637,471.2033 | Fruit of Fiverleaf Akebia |
| 40 | 41.91 | [M+H]+ | 297.1485 | 297.1485 | 1.6 | C19H20O3 | 隐丹参酮 | Cryptotanshinone | 297.1488,282.1259,279.1377,268.1110,251.1440,165.0712 | Salvia miltiorrhiza Bge |

**1. Samples, reagents and instruments**

Huangci granule

Acetonitrile, Ms pure, I09659833, purchased from Merck

Methanol, Ms pure, I0931035804, purchased from Merck

Water, distilled water, 20190430C, purchased from Guangzhou Watsons Food & Beverage Co., Ltd. Beijing branch

Formic acid, Ms pure, 190284, purchased from Thermo Fisher Scientific

Electronic analytical balance，ME104，METTLER TOLEDO

Ultrasonic cleaner，KQ-300 BD, Kunshan Ultrasonic Instrument Co., Ltd

High speed centrifuge，SIGMA 3K15，SIGMA

2. Chromatographic conditions

Instrument:Agilent 1290 UPLC

Chromatographic column:Agilent ZORBAX RRHD SB-C18 (2.1×100 mm, 1.8 µm)

Column temperature:30℃

Injection volume:5 µL

Detection wavelength:254 nm

Mobile phase ratio and flow rate: a phase 0.1% formic acid aqueous solution, B phase acetonitrile, gradient see Table 2.

Table 2 Mobile phase gradient

| Time (min) | Flow Rate (mL/min) | %B |
| --- | --- | --- |
| 0 | 0.3 | 5 |
| 2 | 0.3 | 5 |
| 17  37  43  43.1  45 | 0.3  0.3  0.3  0.3  0.3 | 20  30  95  5  5 |

3. Mass spectrum condition

Instrume: Sciex Triple TOF 4600 LC/MS

Detection mode: ESI离子源Negative/Positive ion mode

Mass spectrum parameters: see Table 3.

Table 3 Mass parameters of Sciex Triple TOF

| MS parameter | Parameter value | MS/MS parameter | Parameter value |
| --- | --- | --- | --- |
| TOF mass range | 50~1700 | MS/MS mass range | 50~1700 |
| Ion Source Gas 1 | 50 | Declustering Potential | 100 |
| Ion Source Gas 2 | 50 | Collision Energy | ±40 |
| Curtain Gas | 35 | Collision Energy Spread | 20 |
| Ion Spray Voltage Floating (kV) | -4500/5000 | Ion Release Delay | 30 |
| Ion Source Temperature (°C) | 500 | Ion Release Width | 15 |
| Declustering Potential | 100 |  |  |
| Collision Energy | 10 |  |  |

4. Preparation of test solution

Take 0.5g of ground sample powder, accurately weigh it, put it into 50ml centrifuge tube, add 30ml of 80% methanol solution to dissolve it, ultrasonic treatment (power 100W, 40KHz) for 30min, cool it to room temperature, shake it up, centrifugation (rotating speed 12000 revolutions per minute) for 5min, take the supernatant, and get it.

Reference

1. Li H, Yao W, Liu Q, et al. Application of UHPLC-ESI-Q-TOF-MS to Identify Multiple Constituents in Processed Products of the Herbal Medicine Ligustri Lucidi Fructus[J]. Molecules, 2017, 22(5):689
2. HPLC-DAD-ESI-Q-TOF/MS法测定金银忍冬花中的化学成分[J]. 中草药, 2017(11).
3. 石慧慧. 复方肠泰颗粒中预知子药材的化学物质基础初探[D]. 2015.
4. 刘文静, 曹妍, 宋青青, 等. 荒漠肉苁蓉花及木质化茎的化学成分定性分析[J]. 中国中药杂志, 2018(18).
5. 祁晓霞, 董宇, 单晨啸,等. 基于UFLC-Q-TOF/MS分析黄芪-丹参药对化学成分研究[J]. 南京中医药大学学报, 2017, 33(33):96.
6. 范姣姣,文红梅,单晨啸,等 基于UFLC-Q-TOF/MS技术的八月札化学成分研究[J]. 中草药, 2013, 44(23):3282-3288.
7. 刘彦玲, 刘传鑫, 田悦, 等. 基于UPLC-Q-TOF/MS的渴络欣胶囊化学成分研究[J]. 药物评价研究, 2019, 42(04):31-38.
8. 李明, 赫军, 马秉智, 等. 中药蛇莓化学成分和抗肿瘤药理作用的研究进展[J]. 中国医院用药评价与分析, 2017(5).
9. 刘量, 叶静, 李萍, 等. 杜鹃兰假鳞茎化学成分研究[J]. 中国中药杂志, 2014, 39(2):250-253.
10. 颜昱. 中药肉苁蓉化学成分及体内成分分析[D]. 2018.
11. 李瑞燕, 赵明波, 屠鹏飞, 等 一标多测法同时测定肉苁蓉中5种苯乙醇苷类成分的含量（英文）[J]. Journal of Chinese Pharmaceutical Sciences, 2019(8).
